# CD4^+^ T-cell DNA methylation changes during pregnancy significantly correlate with disease-associated methylation changes in autoimmune diseases

**DOI:** 10.1080/15592294.2021.1982510

**Published:** 2021-10-04

**Authors:** Tejaswi V. Badam, Sandra Hellberg, Ratnesh B. Mehta, Jeannette Lechner-Scott, Rodney A. Lea, Jorg Tost, Xavier Mariette, Judit Svensson-Arvelund, Colm E. Nestor, Mikael Benson, Göran Berg, Maria C. Jenmalm, Mika Gustafsson, Jan Ernerudh

**Affiliations:** a Bioinformatics Department of Physics, Chemistry and Biology, Linköping University, Linköping, Sweden; b School of Bioscience, Skövde University, Skövde, Sweden; c Division of Inflammation and Infection, Department of Biomedical and Clinical Sciences, Linköping University, Linköping, Sweden; d School of Medicine and Public Health, University of Newcastle, Callaghan, Australia; e Centre for Brain and Mental Health, Hunter Medical Research Institute, New Lambton Heights, Australia; f Department of Neurology, John Hunter Hospital, New Lambton Heights, Australia; g Institute of Health and Biomedical Innovations, Genomics Research Centre, Queensland University of Technology, Kelvin Grove, Australia; h Laboratory of Epigenetics and Environment, Centre National De Recherche En Génomique Humaine, CEA-Institut De Biologie Francois Jacob, Evry, France; i Université Paris-Saclay, AP-HP-Université Paris-Saclay, Hôpital Bicêtre, Institut National de la Santé et de la Recherche Médicale (Inserm) U1184, Center for Immunology of Viral Infections and Autoimmune Diseases, France; j The Centre for Individualized Medicine, Department of Biomedical and Clinical Sciences, Linköping University, Linköping, Sweden; k Department of Obstetrics and Gynaecology and Department of Biomedical and Clinical Sciences, Linköping University, Linköping, Sweden; l Department of Clinical Immunology and Transfusion Medicine and Department of Biomedical and Clinical Sciences, Linköping University, Linköping, Sweden

**Keywords:** Pregnancy, epigenetics, methylation, CD4^+^ T cells, module, rheumatoid arthritis, multiple sclerosis, systemic lupus erythematosus

## Abstract

Epigenetics may play a central, yet unexplored, role in the profound changes that the maternal immune system undergoes during pregnancy and could be involved in the pregnancy-induced modulation of several autoimmune diseases. We investigated changes in the methylome in isolated circulating CD4^+^ T-cells in non-pregnant and pregnant women, during the 1^st^ and 2^nd^ trimester, using the Illumina Infinium Human Methylation 450K array, and explored how these changes were related to autoimmune diseases that are known to be affected during pregnancy. Pregnancy was associated with several hundreds of methylation differences, particularly during the 2^nd^ trimester. A network-based modular approach identified several genes, *e.g., CD28, FYN, VAV1* and pathways related to T-cell signalling and activation, highlighting T-cell regulation as a central component of the observed methylation alterations. The identified pregnancy module was significantly enriched for disease-associated methylation changes related to multiple sclerosis, rheumatoid arthritis and systemic lupus erythematosus. A negative correlation between pregnancy-associated methylation changes and disease-associated changes was found for multiple sclerosis and rheumatoid arthritis, diseases that are known to improve during pregnancy whereas a positive correlation was found for systemic lupus erythematosus, a disease that instead worsens during pregnancy. Thus, the directionality of the observed changes is in line with the previously observed effect of pregnancy on disease activity. Our systems medicine approach supports the importance of the methylome in immune regulation of T-cells during pregnancy. Our findings highlight the relevance of using pregnancy as a model for understanding and identifying disease-related mechanisms involved in the modulation of autoimmune diseases.

**Abbreviations**: BMIQ: beta-mixture quantile dilation; DMGs: differentially methylated genes; DMPs: differentially methylated probes; FE: fold enrichment; FDR: false discovery rate; GO: gene ontology; GWAS: genome-wide association studies; MDS: multidimensional scaling; MS: multiple sclerosis; PBMC: peripheral blood mononuclear cells; PBS: phosphate buffered saline; PPI; protein-protein interaction; RA: rheumatoid arthritis; SD: standard deviation; SLE: systemic lupus erythematosus; SNP: single nucleotide polymorphism; T_H_: CD4^+^ T helper cell; VIStA: diVIsive Shuffling Approach.

## Introduction

Successful pregnancy is a tightly and timely coordinated dynamic immunological process, where the maternal immune system adapts to the presence and the needs of the growing semi-allogenic foetus [1]. How the maternal immune system balances the opposing needs of both promoting tolerance, whilst at the same time maintaining effective immunity against invading pathogens, is not fully understood. The immunological adaptations are most pronounced locally at the foetal-maternal interface, but significant systemic immune alterations also take place [2]. Failure to properly regulate immune responses is associated with several pregnancy complications [3]. Interestingly, pregnancy has also been shown to transiently modulate the disease activity of several autoimmune diseases. In particular, multiple sclerosis (MS) and rheumatoid arthritis (RA) are diseases that may significantly improve during pregnancy [4,5], whereas other diseases such as systemic lupus erythematosus (SLE) may worsen [6]. Thus, pregnancy could serve as a model that offers novel insights for understanding the immune regulatory mechanisms that underlie the transient modulation of autoimmune diseases.

CD4^+^ T helper (T_H_) cells are central in immune regulation and decisive for the balance between immunity and tolerance. The need for regulating T_H_ cell responses is evident by the close association between T_H_ cells and several autoimmune diseases including MS, RA and SLE [7]. Furthermore, the relative lack of T_H_ cells and the increased proportion of regulatory T cells at the foetal-maternal interface [8–10], also support the importance of regulating T_H_ cells during pregnancy. Indeed, dysregulated T_H_ cell responses have been implicated in several pregnancy complications such as preeclampsia, preterm birth and spontaneous recurrent miscarriages [11–17].

Epigenetic modifications are a key avenue for controlling T_H_ cell responses, including methylation of DNA CpG dinucleotides and post-translational histone modifications [18]. Epigenetic modifications can change gene and subsequent protein expression without altering the underlying DNA sequence, thus allowing for a rapid adaptation of cells to the surrounding environment. For example, exposure to cytokines during T_H_ cell differentiation induces epigenetic changes that promote T_H_ cell lineage commitment, tailored towards the nature of the invading pathogens [18]. Epigenetics also provides an attractive, yet unexplored, mechanism for immune regulation of T_H_ cells during pregnancy, which could help explain how T_H_ cell responses are able to adapt and respond to the dynamic changes throughout pregnancy. Interestingly, epigenetic alterations have been implicated in several autoimmune diseases. In SLE, methylation changes in T cells are correlated with disease activity [19], and in MS, several regions in T_H_ cells are differentially methylated in patients compared to controls [20]. However, it is not known if pregnancy specifically affects methylation patterns associated with autoimmune diseases.

In the present study we aimed to investigate the global methylation changes that occur during pregnancy to *(1)* increase our understanding of the adaptations that occur in T_H_ cells during pregnancy and *(2)* assess if these methylation changes play a role in the pregnancy-associated modulation of autoimmune diseases. We therefore examined DNA methylation levels in non-pregnant women and at different time points during pregnancy, and found prominent changes in methylation levels, involving several hundreds of genes, during the second, but not the first, trimester of pregnancy. In order to prioritize among those genes, we used protein-protein interactions (PPI) and four relevant disease module-defining methods [21] and identified a pregnancy methylation module comprising 69 highly interconnected genes. Interestingly, this module was found to be significantly enriched for disease-associated methylation changes for MS, RA and SLE and the directionality of those changes was in accordance with the expected effect of pregnancy on the disease activity. In summary, this systems medicine approach reveals that changes in methylation could mechanistically be involved in the dampening and worsening of autoimmune diseases during pregnancy.

## Materials and methods

### Study population and blood sampling

Peripheral blood was collected from non-pregnant healthy women (n = 12; median age 27 years, range 22–31), 1^st^ trimester (n = 11; median age 25 years, range 16–42) and 2^nd^ trimester (n = 12; median age 32 years, range 24–37) pregnant women. The 1^st^ trimester blood samples were collected from women undergoing elective surgical abortions at Linköping University Hospital. All pregnancies were confirmed viable by ultrasound and the median gestational age was 9 weeks, range 7–10, as determined by crown-lump length measurement. The 2^nd^ trimester pregnant women were recruited at an outpatient maternal clinic in Motala, Linköping University Hospital, and blood samples were obtained at median gestational week 24, range 24–25. The non-pregnant controls were recruited amongst students and personnel at Linköping University and University Hospital and did not take any hormonal contraceptives or other medications. The obstetrical history of the participants is summarized in Table 1. Informed consent was obtained prior to sampling and in one case parental consent was obtained, as the donor’s age was less than 18 years. The study conforms to the Declaration of Helsinki and was approved by the regional ethics committee in Linköping, Sweden (ethical permit number: M39–08).

**Table 1.** Information about the participating women.

		Pregnant	
	Healthy non-pregnant women	1^st^ trimester	2^nd^ trimester
Number of subjects (n)	12	11	12
Age at inclusion (yrs, range)	27 (22–31)	25 (16–42)	32 (24–37)
Use of hormonal contraceptives (yes/no)	0/12	N/A	N/A
Phase of the menstrual cycle (Follicular/Luteal)^a^	3/5^b^	N/A	N/A
Current pregnancy			
Gestational week at inclusion	N/A	9 (7–10)	24 (24–25)
Week of delivery	N/A	N/A	38 (35–41)
Sex of baby (male/female)	N/A	N/A	6/5 ^c^
Birth weight (g)	N/A	N/A	3510 (2320–4375)^c^
Birth method (PN/VE/CS)	N/A	N/A	8/1/3
Pregnancy history			
Previous pregnancies (n)	0 (0–0)^b^	2 (0–10)	1 (0–4)
Previous births (n)	0 (0–0)^b^	1 (0–5)	1 (0–2)

Data is shown as median and ranges (in parenthesis) or as categorical data

^a^based on a 28-day menstrual cycle

^b^Missing data for four non-pregnant women

^c^Missing data from one 2^nd^ trimester pregnant women

N/A, not applicable; PN, normal delivery; VE, vacuum extraction; CS, caesarean section

## CD4^+^ T-cell isolation from peripheral blood mononuclear cells

**Figure 1.**
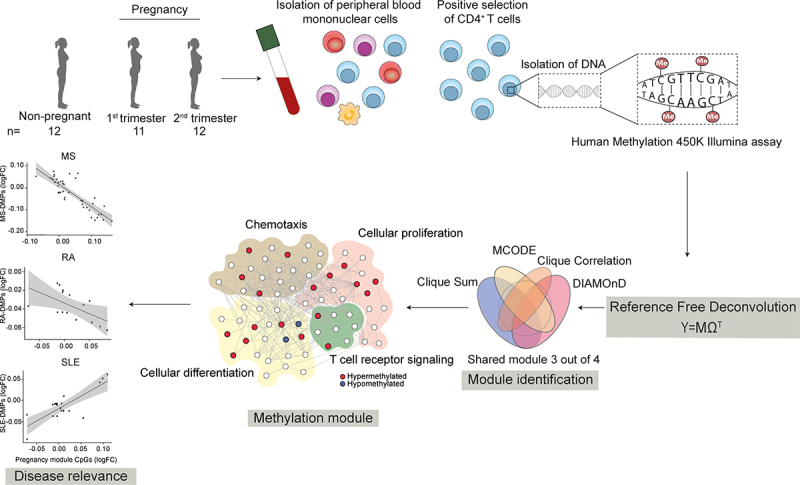
**Overview of the study**. CD4^+^ T cells were isolated from peripheral blood samples collected from 1^st^ and 2^nd^ trimester pregnant and non-pregnant controls. DNA was extracted and genome-wide profiling of DNA methylation was performed using the Illumina 450K array. Reference-free deconvolution was used prior to bioinformatic analysis. A pregnancy module was identified as genes shared between at least three out of the four methods used. The identified shared module, based on differentially methylated genes between 2^nd^ trimester pregnant as compared to non-pregnant women, was interrogated for disease relevance using methylation data of CD4^+^ T cells from multiple sclerosis (MS), rheumatoid arthritis (RA) and systemic lupus erythematosus (SLE).

Venous blood was collected in sodium-heparin tubes (Greiner Bio-One, Kremsmünster, Austria) and peripheral blood mononuclear cells (PBMCs) were isolated by density centrifugation over a Lymphoprep gradient (Axis-Shield, Oslo, Norway) and washed thrice in Hank’s Balanced Salt Solution (Gibco™, Thermo Fisher Scientific, Waltham, MA, USA). The PBMCs were resuspended in MACS buffer (phosphate buffered saline (PBS; Medicago, Uppsala, Sweden) supplemented with 2 mM EDTA (Sigma-Aldrich, St Louise, MO, USA) and 5% foetal bovine serum (FBS; Thermo Fisher Scientific) and labelled with anti-CD4-coated microbeads (Miltenyi Biotech, Bergish Gladbach, Germany). The CD4^+^ cells were isolated by positive immunomagnetic selection using MS columns and a miniMACS separator (Miltenyi Biotec) according to the manufacturer’s instructions. An overview of the experimental study design is shown in Figure 1.

## Flow cytometry analysis and gating strategy

A portion of the cells was used for flow cytometric analysis of purity and the proportion of naive and memory T_H_ cells. The cells were resuspended in PBS+0.1% FBS and labelled with mouse anti-human CD4 (PerCP or PE, clone SK3), CD45RA (FITC, clone L48 or V540, clone HI100), CD45RO (FITC or PeCy7, clone UCHL1) or isotypes IgG1 (FITC, clone X40) and IgG2 (PeCy7, clone G155–178). All antibodies were purchased from BD Bioscience (Franklin Lakes, NJ, USA). Data was acquired using a BD FACS Canto II flow cytometer (BD Biosciences) and analysed using Kaluza software version 2.1 (Beckman Coulter, Brea, CA, USA). The lymphocytes were gated according to forward and side scatter characteristics and defined as naive (CD45RA^+^) or memory (CD45RA^−^) CD4^+^ cells (For gating strategy see Figure S1). The purity of the CD4^+^ cells after isolation was 96.5%±7.5 (mean ± standard deviation (SD)) for non-pregnant, 95.1%±4.5 for 1^st^ trimester and 98.6%±0.7 for 2^nd^ trimester pregnant women. Statistical differences in cell populations were determined using one-way ANOVA and Tukey’s multiple comparisons test using GraphPad version 8.3.0 (GraphPad Software Inc., San Diego, CA, USA). A p-value <0.05 was considered statically significant. Flow cytometry data is shown as mean ± SD.

## Genomic DNA extraction

DNA was extracted using an organic extraction method. Briefly, isolated CD4^+^ cells were washed in ice-cold PBS (Medicago) and incubated in digestion buffer consisting of 100 mM NaCl, 10 mM Tris-HCI (pH 8), 25 mM EDTA (pH 8), 0.5% sodium dodecyl sulphate and 0.1 mg/mL proteinase K overnight at 50°C on a shaker. Phenol-Chloroform-Isoamyl alcohol (ratio 25:24:1) was added and the sample centrifuged. The aqueous phase was transferred and mixed with 3 M sodium acetate, 100% ethanol and GlycoBlue™ (ThermoFisher Scientific). The pellet was air-dried and then dissolved in Tris-EDTA buffer (10 mM Tris-HCI). The DNA was quantified using the Picogreen™ dsDNA Assay Kit (Invitrogen, Carlsbad, CA, USA) and stored at −70°C.

## Illumina 450K methylation assay

For the global DNA methylation assay on >485,000 individual CpG sites, the Infinium Human Methylation 450k bead chip (Illumina, San Diego, CA, USA) was used. The methylation analysis was performed at the SNP&SEQ Technology platform at SciLifeLab (Uppsala University, Uppsala, Sweden). 500 ng of genomic DNA was bisulphite converted using the EZ-96 DNA Methylation Gold™ Kit (Zymo Research, Irvine, CA, USA). Samples from the different groups were distributed across the array slides to limit potential batch effects. Quality controls were performed on GenomeStudio Software 2011.1 (Illumina) as per the manufacturer’s instructions.

## Pre-processing of array data

Data from the Illumina Human Methylation 450K array was analysed in the R programming environment (R version 3.6.1; R Foundation). Preparation and preprocessing of the raw data was performed using the Chip Analysis Methylation Pipeline (ChaMP; version 2.18.0) [22]. The filtering included removing probes *(1)* with detection p-value >0.01 (n = 3732); *(2)* with a bead count <3 (n = 1346); *(3)* without GC start (n = 2924); *(4)* where the probed CpG occur near a single nucleotide polymorphism (SNP) (n = 58,458) and *(5)* where the probe aligned to multiple locations (n = 11). A total number of 419,041 probes were retained after filtering. Since all samples were from female subjects, no filtering of probes on the X chromosome was performed. The filtered raw methylation data were normalized using the Beta-Mixture Quantile dilation (BMIQ) normalization method [23].

In order to identify the number of cell subtypes in the total CD4^+^ T cells, a reference-free deconvolution method based on the work of Houseman et al. 2016 [24] was applied. The RefFreeEWAS package (version 2.2) in R was used on the normalized beta methylation matrix. The reference free deconvolution for cell proportions uses the non-negative matrix factorization and the resulting omega values are used as for identifying the differentially methylated probes (DMPs).

## Differential methylation analysis

DMPs were identified using linear mixed models, logit(Y) = Xß0+ Ωß1+∈ (limma version 3.42.0) [25], where Y is the known methylation beta matrix, X and Ω are the designs relating the observations Y to ß0 and ß1 respectively and ∈ is the random error. Design X is the vector of the fixed effects (non-pregnant, 1^st^ Trimester and 2^nd^ Trimester) and Ω is the design matrix with deconvoluted cell proportions of the respective samples. Due to the bimodal distribution of beta values, the matrix was logit transformed to use M values for application of the covariates in the model. The delta beta was calculated as the difference between the mean beta values of the two comparisons. The p-values were corrected for multiple testing using false discovery rate (FDR) correction. Probes that were considered significantly differentially methylated had an FDR corrected p-value <0.05 and also had a beta methylation difference of ± 5% (Table S1 and S2) between the two comparisons group.

Multidimensional scaling (MDS) was performed using the mdsPlot function from the package minfi in R [26]. Differentially methylated genes (DMGs) were identified by mapping the ranked list of DMPs to genes and genomic position (position, chromosome, strand and additional features), using the probe information in the IlluminaHumanMethylation450Kanno.ilmn12.hg19 package [27]. Only genes that had at least one DMP were then selected as DMGs. In case of multiple DMPs mapping to a single gene, a gene was considered hypermethylated if a majority of DMPs showed an increase in methylation delta beta value of more than 5% with the highest FDR corrected p-value, and *vice versa* in case of hypomethylation.

## Module identification

To get a functional understanding of the methylation changes induced by pregnancy, the DMGs from the comparison between 2^nd^ trimester pregnant and non-pregnant women (*i.e.*, the time points with the biggest difference) were used as input for estimating modules of the gene network. We used four different module algorithms (MCODE [28], Correlation Clique [29], Clique Sum [30], DIAMOnD [31]) from our previously published R package MODifieR version 0.1.3 [21], because they are integrating known gene-gene interactions and have been shown to work with huge number of probes. The underlying network of gene-gene interactions was derived from the publicly available protein-protein interaction database STRING (version 11) [32] by including only interactions with a combined evidence score of at least 900. We used a consensus approach, such that the final module comprised the union of genes included in modules from at least three out of the four methods. The number of genes in the modules produced by each method were: n = 346 (DIAMOnD), n = 3319 (MCODE), n = 451 (Clique Sum) and n = 76 (Correlation Clique) (Table S3). Visualization of the network was done in Cytoscape (version 3.7.2) [33].

## Analysis of disease relevance of the pregnancy module

The resulting pregnancy module was interrogated for disease relevance using methylation data set from three different autoimmune diseases (MS [34], SLE and RA (Tost, Mariette *et al*., unpublished)), whose disease activity have been shown to change during pregnancy. Only female samples were included for the identification of DMPs between patients and controls for the different diseases. Previous analyses of the MS dataset reported no genome-wide significant probes [20]. However, an unsupervised inspection using multidimensional scaling suggested that this was due to partially overlapping sub-groups of patients and controls (Figure S2). As our intention was to investigate changes in the large-scale pathogenic profiles rather than identifying new diagnostic biomarkers, we identified sample subgroups with clear large-scale differences using the diVIsive Shuffling Approach (VIStA) [35]. This approach is based on identifying groups with the maximal differences in methylation expression while excluding overlapping sub-groups of samples from different phenotypes. We identified the sample sub-groups that showed clear large-scale differences for all three diseases. The total number of samples before VIStA: MS, n = 28 patients, 26 controls; RA, n = 22 patients, 33 controls; SLE, n = 23 patients, 33 controls and after VIStA: MS, n = 28 patients, 10 controls; RA, n = 22 patients, 23 controls and SLE, n = 23 patients, 26 controls (Figure S2). The enrichment of disease genes in the DMP-derived genes identified by VIStA was done by Fisher’s exact test using known disease genes derived from DisGeNET [36] (n = 1832 for RA, n = 1112 for SLE and n = 1117 for MS). We computed the Spearman and Pearson correlations between the pregnancy module CpGs from the module and the disease-associated DMPs, p-values lower than 0.05 were considered statistically significant.

## Functional enrichment analysis

At several different steps in the analysis, pathway enrichment analysis was performed to investigate the biological relevance of the identified genes. The pathway enrichment analysis was based on gene ontology (GO) using *enrichGO* from clusterProfiler (version 3.14.3) [37]. An adjusted p-value ≤ 0.05 (Benjamini-Hochberg) was considered statistically significant.

## Results

### CD4^+^ T cells undergo methylation changes in several hundred CpG sites during pregnancy

**Figure 2.**
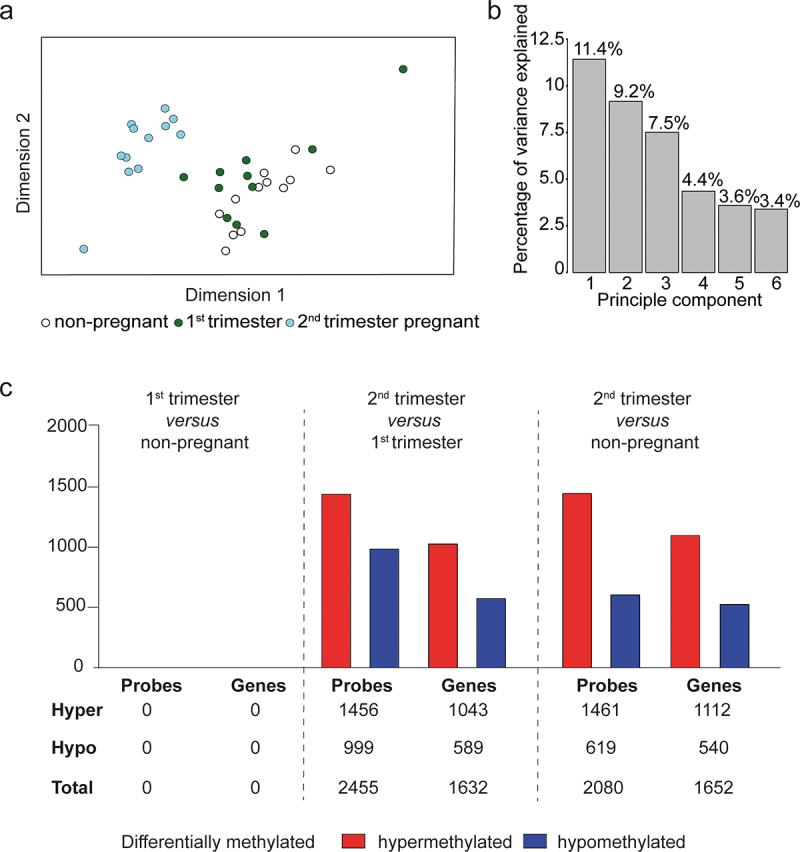
**Differential methylation in CD4^+^ T cells during pregnancy**. (a) Classical multidimensional scaling of global methylation data obtained from isolated CD4^+^ T cells from 1^st^ (green circles; n = 11) and 2^nd^ (blue circles; n = 12) trimester pregnant and non-pregnant (unfilled circles; n = 12). (b) Bar graph of the percentage of variance explained by the first six principal components. (c) Differential expression of methylated probes and genes between pregnant and non-pregnant women using a cut-off of FDR<0.05 with an MMD < −0.05 and > 0.05. Hypermethylated probes and genes are shown in red and hypomethylated probes are shown in blue. The data have been processed for filtering, BMIQ-normalization and adjusted for cell-type heterogeneity using reference-free deconvolution by Houseman et al. (2016), adjusting for two major cell types. BMIQ: beta mixture quantile dilation, FDR: false discovery rate, MMD: mean log2 methylation difference.

To assess changes in the methylation patterns induced during pregnancy, we performed whole genome DNA methylation profiling of isolated CD4^+^ T cells from 1^st^ (n = 11) and 2^nd^ (n = 12) trimester healthy pregnant and from non-pregnant women (n = 12). An overview of the study is shown in Figure 1. To obtain an initial global assessment of the methylation differences within and across the groups, we performed unsupervised clustering using classical multidimensional scaling (MDS), which revealed clear differences between 2^nd^ trimester and 1^st^ trimester pregnant women as well as between 2^nd^ trimester and non-pregnant women, whereas no clear differences were evident between 1^st^ trimester and non-pregnant women (Figure 2a). Moreover, we found similar variance explained by each of the first three components (7.5–11.4%), while the remaining components explained less than 4.4% of the variance each (Figure 2b), suggesting that there is not one dominating variable that contributes to the variance but rather several, discrete factors that are driving the observed changes. The differences between the groups became apparent only after adjusting for the cell-type composition in the samples by using reference-free deconvolution [24]. The rationale behind using reference-free deconvolution was based on the observed changes in the proportion of naive and memory cells during pregnancy (Figure S3a). Since differences in methylation patterns between naive and memory cells may be a confounder [38], we decided to adjust for cell-type composition. We therefore calculated the proportions of presumed cell types in the samples and selected the optimal number of cell types (*K* = 2) as this was the number of cell types that minimized the variance of the bootstrapped deviance matrix (Figure S3b). The inferred proportions of memory T cells were found to be highly correlated with the actual proportions of this subset based on flow cytometry analysis (R = 0.68, p = 2.7x10^−5^; Figure S3c), thus supporting the use of reference-free deconvolution as a method for accurately inferring cell proportions. Furthermore, the deconvolution approach based on the presence of two major cell types fits with the view of naive and memory cell proportions accounting for the major differences in methylation patterns as no differences were found between groups when not taking this into consideration (Figure S4a). This observation was also supported by the fact that a much larger part of the variance was explained by the first principal component (26.8%; Figure S4b) as compared to when using reference-free deconvolution. The deconvolution approach was also needed in order to enable inclusion of other data sets where evaluation of cell proportions have not been performed.

In agreement with the MDS, differential methylation analysis showed many significantly DMPs and DMGs between 2^nd^ and 1^st^ trimester and between 2^nd^ trimester and non-pregnant women, whereas no differences were found between 1^st^ trimester and non-pregnant women (FDR<0.05, 5% log2fold change (logFC) threshold for methylation change; Figure 2c and Table S1 and S2). Overall, it seems like pregnancy is characterized by an increase in methylation of CD4^+^ T cells, as evident by a more than 2-fold increase in hyper- as compared to hypomethylated DMPs during 2^nd^ trimester (as compared to non-pregnant; 67% vs. 33%). Similarly, more DMPs were hypermethylated comparing 2^nd^ and 1^st^ trimester pregnancy (59% vs. 41%). To gain insights into the biological relevance of the observed methylation changes, we performed pathway analysis on the DMGs using GO. Quite surprisingly, no immune-related pathways were found among the DMGs for any of the comparisons (Figure S5a-b). Instead, non-immune related pathways like cellular development, organization and differentiation were over-represented among the significant pathways.

## Using a network-based modular approach reveals methylation changes in genes related to T-cell signalling and T-cell activation

It is known that disease-related genes tend to co-localize in the PPI network and form modules of functionally related genes, *i.e.*, genes belonging to the same biological processes [39]. We here focused on the DMGs from the comparison between 2^nd^ trimester and non-pregnant healthy women to identify changes that are induced by pregnancy and examined if these genes were also co-localized, which could suggest functional relationships. For this, we used all high confidence human protein-protein interactions (n = 331,219) from among 12,465 proteins (STRING version 11) [32] and computed the distance between our pregnancy DMP-derived genes in the PPI network. We found that the DMP-derived genes had a significantly shorter distance, compared to randomly equally sized sets of proteins (harmonic average <d_pregnancy_≥ 3.60 versus <d_random_≥ 3.98; permutation test p = 0.03), where distance is defined as the smallest number of interacting proteins between two given genes. Thus, the pregnancy DMP-derived genes appear to be co-localized, indicating a functional relationship in similarity to what has previously been observed for disease-related genes.

**Figure 3.**
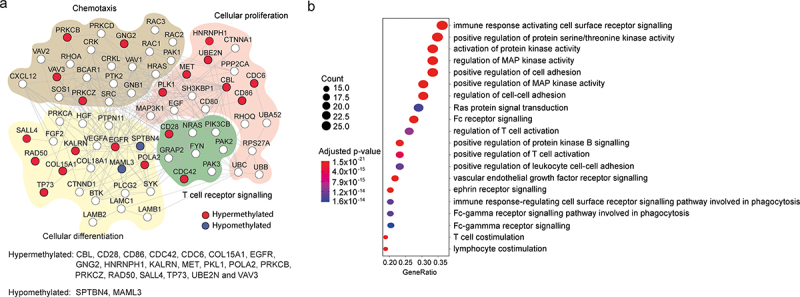
**Module of methylation changes induced during pregnancy**. (a) Graphical illustration of the module identified based on the genes derived from the DMPs (FDR<0.05). Nodes represent genes and the connecting lines protein-protein interactions. Red nodes correspond to the genes containing only hypermethylated DMPs and blue nodes only hypomethylated DMPs that were differentially methylated in the original data. Unfilled nodes represent novel genes from the interaction network. The interactions were chosen from the STRINGdb (threshold >0.9). The graphical illustration was constructed without using any threshold for illustrative purposes only. (b) Dot plot of the top 20 most significantly enriched GO pathways based on the 69 genes derived from the module. The x-axis represents the gene ratio, dot size = gene count and dot colour = adjusted p-values. DMPs: differentially methylated probes, FDR: false discovery rate, GO: gene ontology.

This motivated us to apply a network-based modular approach to identify more functionally related genes. We therefore applied a selection of different disease module estimation methods (Clique Sum, Correlation Clique, DIAMOnD and MCODE) on the previously defined DMGs using the R-package MOdifieR [21]. Briefly, these methods combine the DMGs with the PPI network to identify the most interconnected subnetworks comprising the DMGs and additional other tightly interconnected genes. The genes that were picked up by at least three of the four methods were included in a moderately sized pregnancy module comprising 69 genes. Out of these 69 genes each method picked up a minimum of 12 genes (Correlation Clique) and a maximum of 69 genes (Clique Sum). We found that 22 (32%) of the module genes originated from the set of DMP-derived genes whereas the rest comprised genes derived from the interaction network (Figure 3a). Of these 22 genes, 20 were hypermethylated (*e.g., CD28, CD86, PRKCB, TP73, PLK1 and VAV3*) and two were hypomethylated (*SPTBN4, MAML3*) when comparing 2nd trimester pregnant and healthy non-pregnant women. The pregnancy module showed overall enrichment for immune-related pathways, including several pathways related to T-cell activation such as regulation of T-cell activation, T-cell and lymphocyte co-stimulation (Figure 3b and Table S4). Furthermore, several pathways related to kinase regulation were also enriched. Given the role of kinase regulation in T-cell proliferation and differentiation [40], this observation further highlights T-cell regulation as a central process affected by methylation changes during pregnancy. A closer examination of the module showed four major clusters of genes within the module related to: cellular proliferation, chemotaxis, cellular differentiation and T-cell receptor signalling (Figure 3a). Thus, using a modular approach to investigate methylation changes induced during pregnancy highlighted several pathways of potential importance for immune modulation during pregnancy.

## Methylation changes induced during pregnancy correlate with changes observed in autoimmune diseases that are modulated during pregnancy

**Figure 4.**
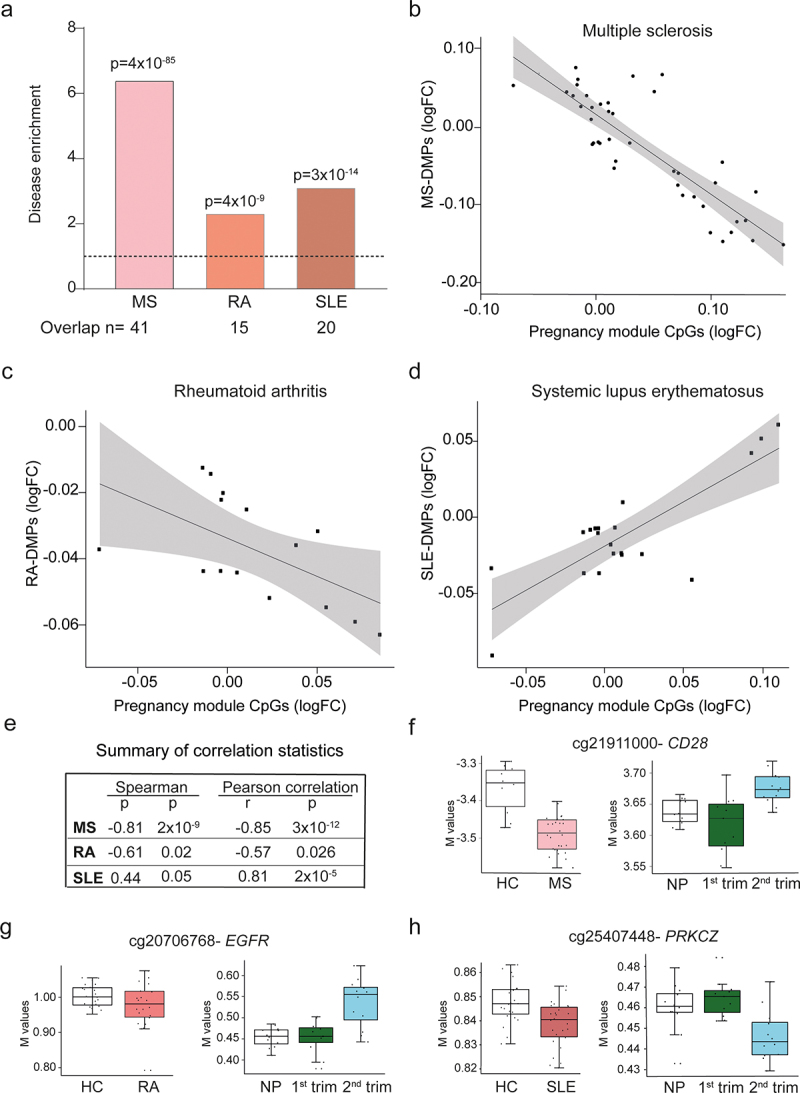
**Correlation between pregnancy-associated and disease-associated methylation changes**. DMPs from three different chronic inflammatory diseases (MS, RA and SLE), that are known to be modulated pregnancy, were generated using Vista [35] (see *Materials and Methods* for more details) and compared to the CpGs from the pregnancy module (FDR<0.05). (a) Fold enrichment of disease-associated DMPs among the CpGs derived from the pregnancy module was calculated using Fisher’s exact test. Number of overlapping disease-associated and pregnancy module CpGs are given below the x-axis for each of the diseases respectively. Correlations between the pregnancy-module CpGs and (b) MS-associated DMPs, (c) RA-associated DMPs and (d) SLE-associated DMP were done by Spearman and Pearson (e). M-values for cg21911000-*CD28* (f), cg20706768-*EGFR* (g) and cg25407448-*PRKCZ* (h) comparing disease versus healthy control and non-pregnant (n = 12), 1st (n = 11) and 2nd trimester (n = 12) pregnant women. For MS: n = 28 patients, n = 10 healthy controls, RA: n = 22 patients, n = 23 healthy controls and SLE: n = 23 patients, 26 controls. The grey shaded area shows 95% confidence interval. DMPs: differentially methylated probes, FDR: false discovery rate, HC: healthy controls, MS: multiple sclerosis, RA: rheumatoid arthritis, SLE: systemic lupus erythematosus, VIStA: diVIsive Shuffling Approach.

The disease activity of several autoimmune diseases is known to be modulated during pregnancy [4–6]. Moreover, our results show that methylation changes are prevalent during pregnancy. We therefore decided to investigate if our identified pregnancy module and related methylation changes was associated with diseases affected by pregnancy. To this end, we used methylation data of CD4^+^ T cells from MS [34], RA and SLE (Tost, Mariette *et al*., unpublished). Finding DMPs in complex diseases that display large disease heterogeneity such MS, has previously been challenging [20]. Indeed, initial analysis of the expression data showed no clear separation between patients and controls for any of the diseases (Figure S2). We therefore used VIStA [35], which is based on identifying subgroups of samples that display the largest difference in methylation profile, hence maximizing the differences between groups. Implementing this approach, we identified distinct groups of patients and controls that showed the largest methylation differences (Figure S2). To validate that the DMPs identified using VIStA were actually relevant to disease, we used disease-associated genes from DisGeNET [36] and found that indeed, the DMP-derived genes were significantly enriched for disease-related genes (MS: fold enrichment (FE) = 2.32, Fisher’s exact test p = 4x10^−7^, n = 78; RA: FE = 2.53, p = 2x10^−12^, n = 116; SLE: FE = 2.46, p = 7x10^−14^, n = 139). Next, we used the CpG sites derived from the pregnancy module, hereon referred to as pregnancy module CpGs, to investigate if these were related to the identified methylation changes associated with disease. Notably, the pregnancy module CpGs were significantly enriched for differentially methylated sites for all three diseases (MS: FE = 6.43, n = 41, Fisher’s exact test, p = 4x10^−85^; RA: FE = 2.35, n = 15, p = 4x10^−9^; SLE: FE = 3.14, n = 20, p = 3x10^−14^; Figure 4a and Table S5). Out of these, 29% (n = 22 out of 76 disease-associated CpGs, covering to 20 out of the 22 DMGs in the module) originated from the DMPs from the pregnancy module. Furthermore, not only were they significantly enriched but there was also a significant negative correlation between the pregnancy-DMPs and the disease-associated DMPs for MS (Pearson r = −0.85, p = 3x10^−12^; Spearman *rho *= −0.81, 2x10^−9^; Figure 4b) and RA (Pearson r = −0.57, p = 0.026; Spearman *rho *= −0.61, 0.02; Figure 4c). Conversely, for SLE, there was a significant positive correlation (Pearson r = 0.81, p = 2x10^−5^, Spearman *rho* = 0.44, p = 0.05; Figure 4(d–e)). Interestingly, the direction of the correlations is in full agreement with previous findings that women with MS and RA experience a temporary improvement during pregnancy, while disease activity in women with SLE may worsen. This pattern is further highlighted and exemplified in (Figure 4(f–h)) by cg21911000- *CD28* and cg20706768- *EGFR* that are less methylated in MS and RA respectively but increase in methylation during pregnancy in contrast to cg25407448- *PRKCZ* in SLE that was less methylated in both disease and during 2nd trimester pregnancy.

## Discussion

This study represents, to our knowledge, the first global methylation analysis that evaluates the temporal changes induced in circulating CD4^+^ T cells during pregnancy and how these changes relate to autoimmune diseases whose disease activity is altered during pregnancy. We identified several hundreds of genes that were differentially methylated during pregnancy, particularly in the second trimester, emphasizing the systemic changes that occur in CD4^+^ T cells, which could be of importance for controlling alloreactive T cells. Using a network-based modular approach, which highlights the most functionally interconnected genes, disclosed several methylation changes related to T-cell activation (*e.g.*, hypermethylation of *CD28, CD86, VAV3*). Furthermore, the enrichment of differentially methylated sites related to MS, RA and SLE, *i.e.*, diseases known to be affected during pregnancy, indicates an involvement of epigenetic modifications in the temporary improvement and deterioration of these diseases during pregnancy. Importantly, the role of epigenetic contribution to disease activity was further supported by the clear direction of these changes, being in accordance with the directionality of the pregnancy-associated modulation of these diseases.

As the maternal immune system undergoes progressive changes throughout pregnancy [1], we studied methylation patterns at two time points to be able to capture the dynamic changes that occur systemically. Indeed, we found that whereas only modest changes, in comparison to non-pregnancy, were found in the 1^st^ trimester, prominent changes were observed during the 2^nd^ trimester. This is in line with the view that immune regulation takes place mainly locally during the 1^st^ trimester, while the systemic changes are more prominent during the 2^nd^ trimester [41,42]. Interestingly, pregnancy was overall associated with more hyper- than hypomethylated genes. This is in line with an increased regulation of circulating CD4^+^ T cells, in order to limit potentially dangerous alloreactive T cells [14]. Furthermore, the methylation changes could also be central for the T-cell-mediated diseases that are known to improve during pregnancy, where dangerous autoreactive T cells are a driving force in propagating disease. In contrast to our findings on isolated CD4^+^ T cells, whole blood samples showed unchanged [43] or decreased [44,45] levels of methylation during pregnancy. Of note, different arms of the immune responses are differentially regulated during pregnancy, with increase in innate immunity and decrease in T-cell immunity [2] and thus, net methylation levels of these complex changes in blood as a whole tissue might be difficult to interpret. Since methylation changes pertaining to different immune cell populations are not easily captured when assaying whole blood, our approach using isolated CD4^+^ T cells proved advantageous when assessing immune regulatory aspects of pregnancy, especially in relation to T-cell-associated diseases.

Using a network-based approach to analyse the most functionally related pregnancy-associated changes further highlighted genes and pathways involved in T-cell activation and T-cell signalling. Indeed, several sites involved in T-cell activation were found to be hypermethylated during pregnancy. *CD28* is indisputably critical in T-cell activation and in determining the sensitivity of the T cells, thus serving as a crucial checkpoint for T-cell responses. Accordingly, *CD28* has been indicated as an important regulator of autoimmune diseases [46]. *CD86* expression on memory effector T cells has been proposed to serve as a co-stimulator for T-cell responses [47–49] and, thus, it may not only serve as a costimulatory molecule on antigen-presenting cells but also on subsets of T cells. Of note, abatacept, a *CTLA4*-Ig antagonist of the CD80/86-CD28 co-stimulation axis, is a very efficient treatment in RA [50]. Interestingly, *PLK1* was shown to be upregulated on alloreactive T cells in graft-versus-host disease and blocking its expression prevented T-cell activation and induced apoptosis [51]. Further, the pregnancy module also disclosed several other functionally related genes involved in similar processes such as *SYK, FYN, VAV1*, central components in T-cell receptor signalling [52–54]. Notably, T-cell-associated or other immune-related pathways were not apparent from the initial pathway analysis of the differentially methylated genes. In accordance, global methylation analysis of whole blood also revealed mainly presence of metabolic pathways, rather than immunological [44]. Speculatively, the more specific immunological changes might be overshadowed by other non-immune genes also subjected to alterations during pregnancy. However, by using a modular-based approach for a better functional understanding of the genes subjected to methylation changes, we were able to capture these immune-related differences.

Considering the profound effect that pregnancy has on the maternal immune system, it is not surprising that several autoimmune diseases are affected during this time, although the underlying mechanisms are still unknown. Our methylation module of pregnancy included several genes and pathways of interest in terms of autoimmunity. *SYK* has for example been implicated in the SLE pathogenesis by regulating T-cell signalling [55] and is suggested as potential target for treatment of autoimmune diseases [55,56]. *VAV1* plays a central role in controlling immune responses in both MS [57] and RA [58]. Further, several pathways related to Fc receptor signalling were present among the enriched pathways. Although Fc receptor regulation is associated with humoral responses, Fc receptor expression is established to be present on CD4^+^ T cells and binding of IgG is known to promote T-cell activation [59,60]. Interestingly, changes in IgG glycosylation have been linked with the pregnancy-associated improvement in RA [61,62] and thus, although speculatively, our results provides some evidence that changes in Fc-receptor signalling, possibly due to differences in glycosylation patterns during pregnancy, related to CD4^+^ T cells could be involved in the pregnancy-induced improvement of RA. Another module-derived gene *CXCL12* has been associated with both pre-eclampsia and autoimmunity, further supporting the connection between pregnancy- and disease-related changes. Indeed, the pregnancy methylation module was found to be significantly enriched in disease-associated DMPs for MS, RA and SLE. Despite that many non-DMGs were included in the pregnancy module, most of the DMGs that were included were disease-associated, further validating the relevance of the pregnancy-associated changes in terms of diseases. Since the pregnancy module disclosed many genes involved in T-cell signalling and T-cell activation, it might not be surprising that we found an enrichment for these diseases, in particular for MS and RA that are depicted as T-cell mediated diseases. Intriguingly, the observed disease-related changes correlated with the pregnancy-induced changes in accordance with the previously reported effect of pregnancy on the disease activity. For SLE, a positive correlation was found between SLE-associated methylation changes and pregnancy-associated changes in agreement with the temporary worsening of disease during this time. In contrast, for RA and MS, diseases that improve during pregnancy, a negative correlation was found. Indeed, several DMPs displayed this clear pattern of changes. For example, methylation changes in *CD28*, a gene crucial for development of experimental autoimmune encephalomyelitis in mice [63], was less methylated in MS patients as compared to controls but was more methylated in 2^nd^ trimester pregnant as compared to non-pregnant women. Similarly, *EGFR* showed the same opposing pattern in RA related to pregnancy. EGFR has been suggested as a potential treatment for RA, where blockade results in amelioration of disease [64]. Thus, the increased methylation of *EGFR* in pregnancy could be a natural blockade or halting of EGFR activity. Less methylation of the atypical protein kinase C, zeta (gene *PRKCZ*) was observed in SLE patients compared to healthy controls as well as during pregnancy. This is in agreement with pregnancy being a more ‘T_H_2-deviated’ condition, which has been suggested as an explanation why diseases like SLE worsen since PKCζ has been shown to be important in JAK1/STAT6 signalling, thereby controlling T_H_2 effector function [65].

One potential limitation of our study is that, due to the rather large overlap between patients and controls, we had to apply VIStA approach [35] to find disease-associated DMPs. This strategy is similar to the extreme group approach where individuals are selected on the basis of extremes in order to increase statistical power for detecting relationships [66]. Indeed, complex disease often display large disease heterogeneity and detecting differences can be challenging. In accordance, only minor differences in methylation were found in a study of CD4^+^ T cells in patients with MS despite comparing rather large groups of patients and controls [20]. Also, the rather small samples sizes used here makes it even more difficult to identify changes between groups. Thus, the methylation changes described here are the ones that most profoundly separates disease from the healthy state, hence excluding subtle differences that also could be of importance and be subjected by alterations during pregnancy. However, our approach is strengthened by the consistency and correlation of our findings with independent disease data, and we believe that it demonstrates the feasibility of using pregnancy as a model to investigate disease-relevant changes and that future, larger studies can further add to this knowledge.

## Conclusions

Our findings demonstrates that methylation changes of CD4^+^ T cells are prominent at mid-pregnancy, and that these changes could be an important immune regulatory mechanism during pregnancy, contributing to the pregnancy-induced modulation of MS, RA and SLE. Our study describes a new approach for studying disease-associated alterations that could uncover disease mechanisms of importance for future treatment strategies.

## Supplementary Material

Supplemental Material

## Data Availability

The R-code used for data analysis can be found at https://gitlab.com/Gustafsson-lab/pregnancy_methylation_project. The data discussed in this publication have been deposited in NCBI’s Gene Expression Omnibus [67] and are accessible through accession number GSE153459 https://www.ncbi.nlm.nih.gov/geo/query/acc.cgi?acc=GSE153459). Raw and processed data for the RA and SLE sample are available from JT (tost@cnrgh.fr) and XM (xavier.mariette@aphp.fr) upon reasonable request.
