# Supplementary material for: CD4+ T-cell DNA methylation changes during pregnancy significantly correlate with disease-associated methylation changes in autoimmune diseases
Source: Epigenetics. 2021 Oct 4;17(9):1040–55. doi: 10.1080/15592294.2021.1982510 (PMC9487751; doi:10.1080/15592294.2021.1982510)
Supplement: Supplemental Material [file KEPI_A_1982510_SM4016.zip › supplementary/Supplemental Figures_Revised.docx]

**Figure S1. Flow cytometry gating analysis.** Representative plots of flow cytometry analysis for determining purity and phenotyping. Purity of the CD4^+^-sorted cells was assessed by **(a)** gating lymphocytes based on their characteristic forward and side scatter properties and **(b)** on CD4 expression based on CD4 intensity**.** The purity represents the % of CD4^+^ cells. Gating for determining the proportion of naïve (CD45RA^+^) and memory (CD45RA^-^) was based on **(c)** initial gating of lymphocytes according to forward and side scatter and subsequently on **(d)** CD4 expression**. (e)** The CD4^+^ T cells were defined as CD45^+^ and CD45RA^-^ using the contour of the positive and negative populations for CD45RA based on histogram of CD45RA intensity. Two representative samples are shown.

**
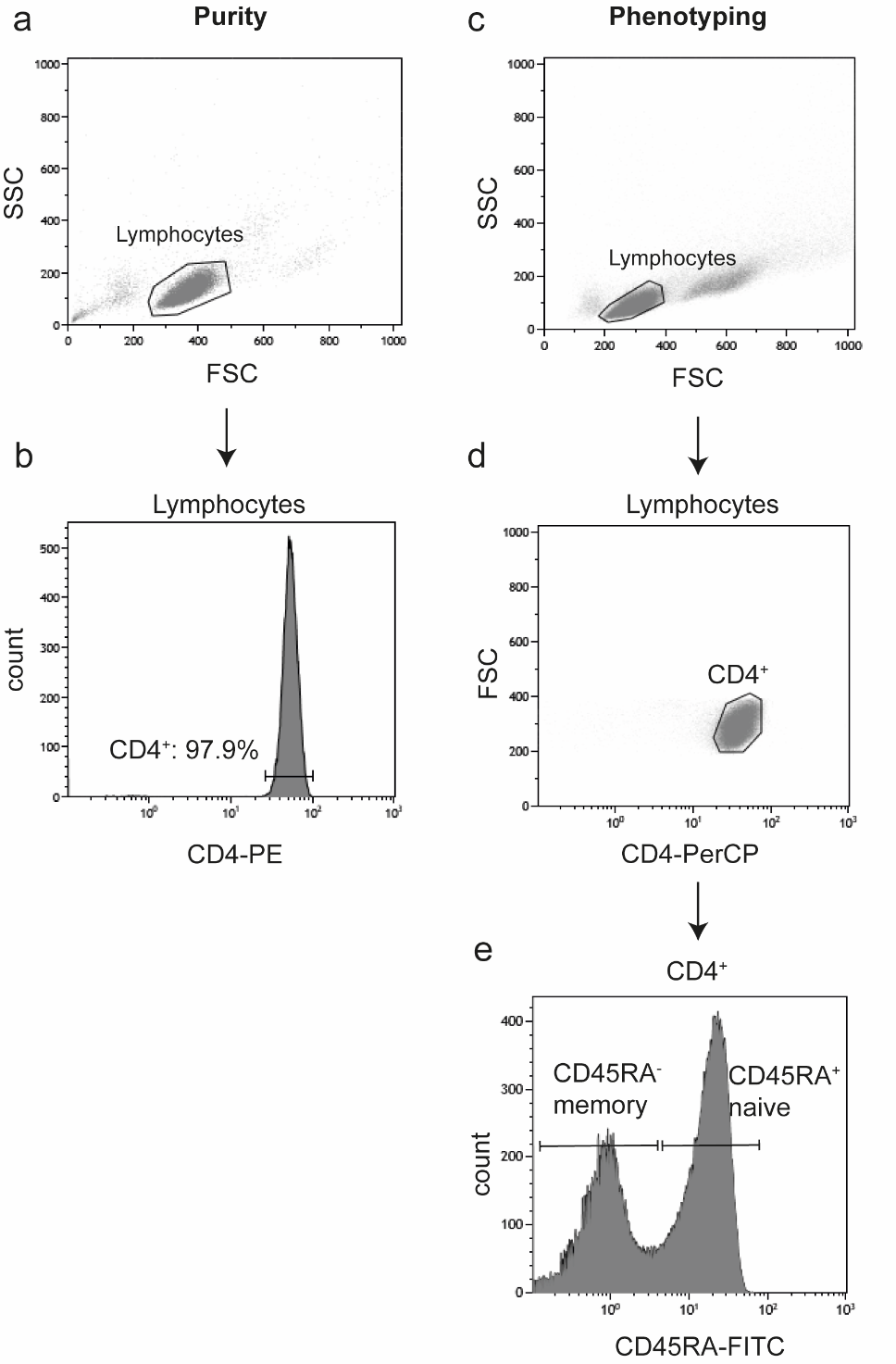
**

**Figure S2. Using the diVIsive Shuffling Approach (VIStA) on methylation data from MS, RA and SLE.** Classical multidimensional scaling (MDS) of methylation changes in CD4^+^ T cells obtained from patients with MS, SLE or RA as compared to healthy controls before and after the diVIsive Shuffling Approach (VIStA) was applied. Only females were included in the analysis. Left vertical panel shows MDS plots before and right vertical panel shows MDS plots after VIStA with patients depicted as open circles and health controls as filled circles. The number of included samples are shown at the bottom left of the plots. For details on how the differential expression analysis was performed see Materials and Methods. MS: multiple sclerosis, RA: rheumatoid arthritis, SLE: systemic lupus erythematosus.


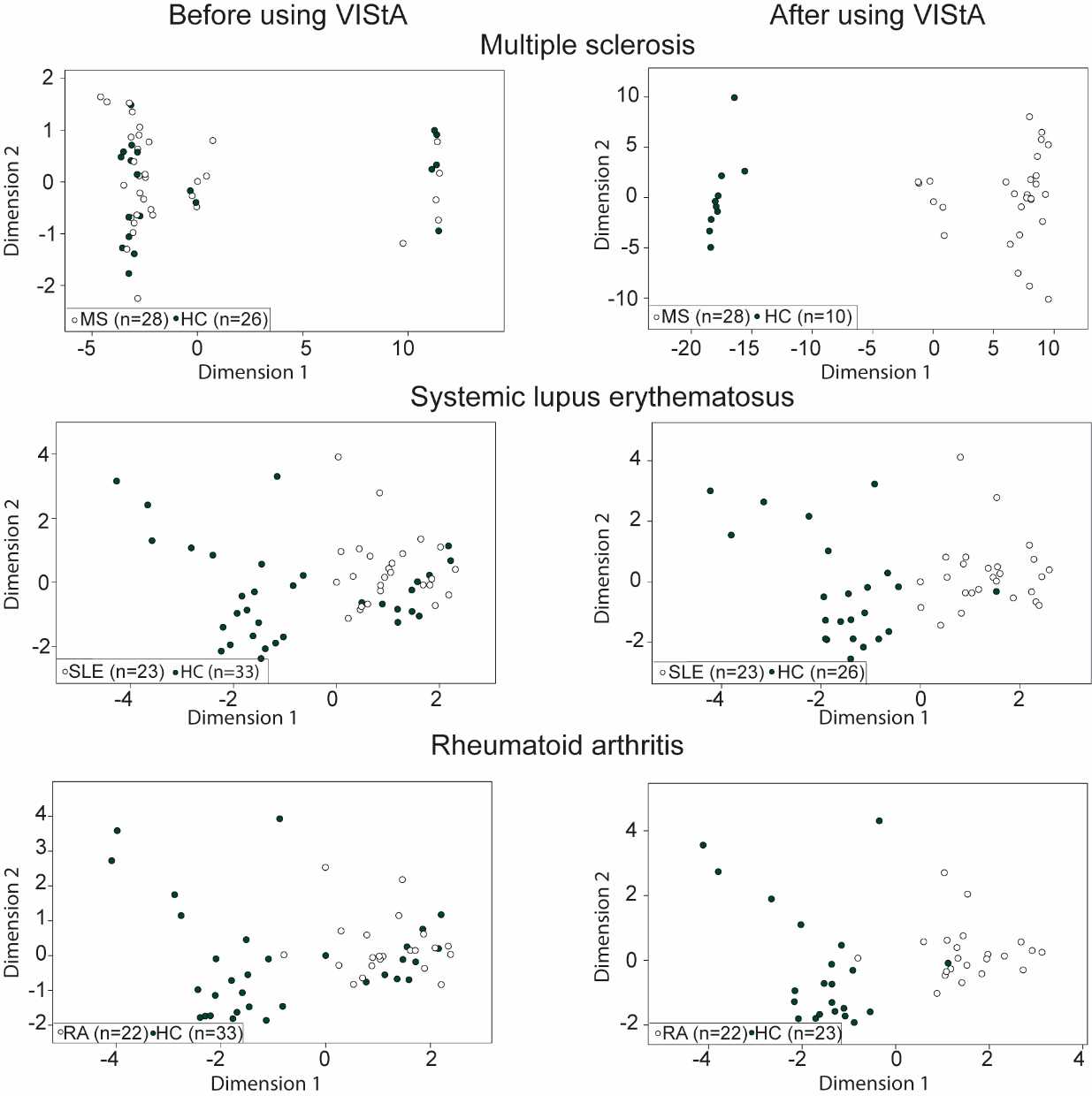


**Figure. S3. Reference-free deconvolution to adjust for cell type heterogeneity. (a)** The proportion of naive (CD45RA^+^) and memory (CD45RA^-^) in non-pregnant (n=12), 1^st^ trimester (n=7) and 2^nd^ trimester (n=12) pregnant women was analysed by flow cytometry. The memory proportion is shown in black bars and the naive proportion in grey. Data regarding the proportion of naive and memory cells is missing for four first trimester pregnant women (n=7 instead of n=11). Data is shown as the percentage of CD4^+^ cells expressing CD45RA, mean+standard deviation. Statistical differences were determined using one-way ANOVA followed by Tukey’s multiple comparisons test. **p≤0.01, ****p≤0.0001. **(b)** Dot plot of the -log deviance when adjusting for one to six different cell types using the reference-free deconvolution according to Houseman et al. (2016). Statistical differences were determined using the permutation test, ***p≤0.001. **(c)** Correlation plot between the memory cell proportions as assessed by flow cytometry compared to those estimated by using reference-free deconvolution. Statistics (rho and p-values) were performed using Pearson correlation test. One dot=one subject and the grey shaded area shows 95% confidence interval.


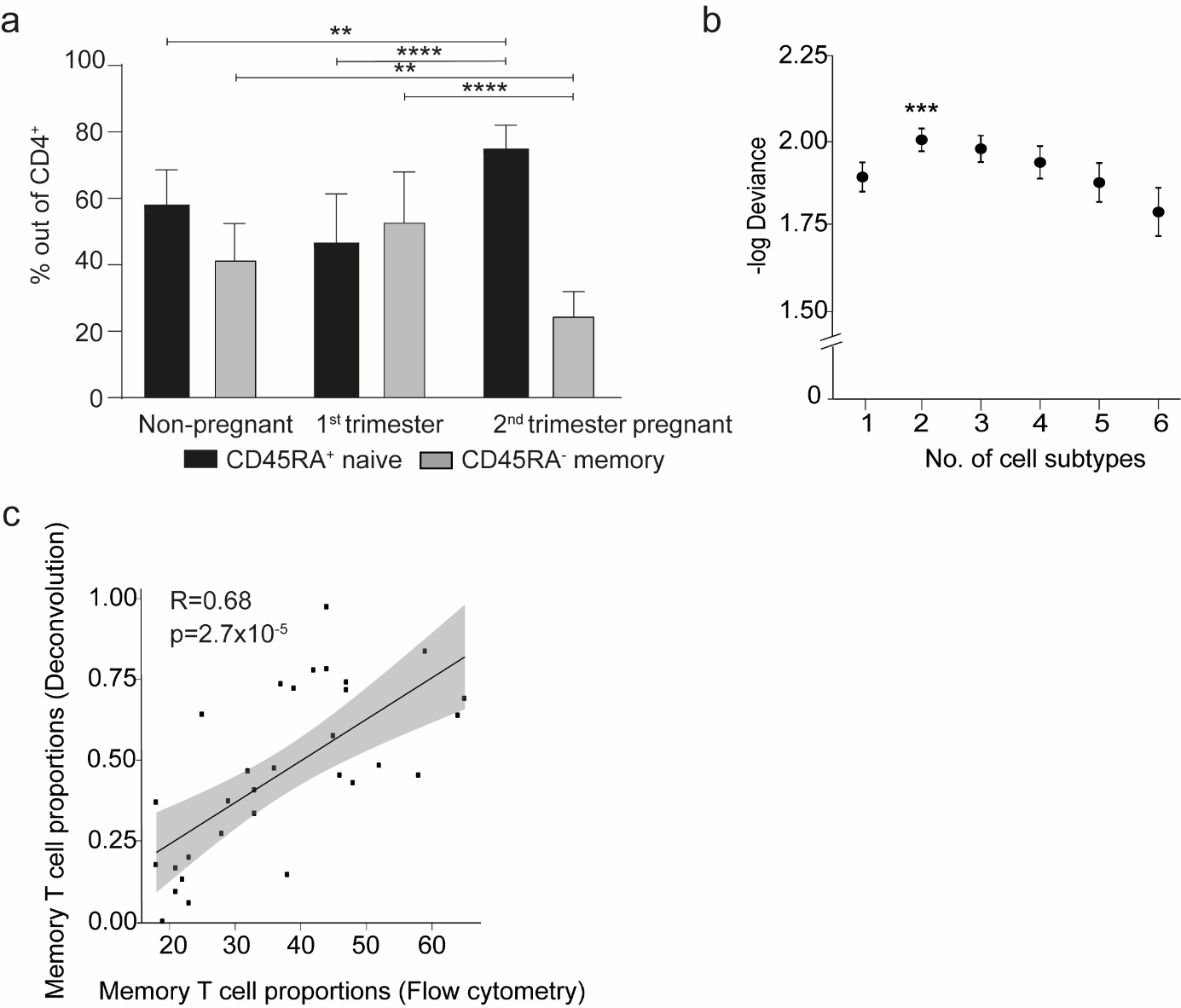


**‘**

**Figure. S4. Methylation changes before reference-free deconvolution.** **(a)** Multidimensional scaling plot of global methylation changes obtained from isolated CD4^+^ T cells from 1^st^ (green circles; n=11) and 2^nd^ (blue circles; n=12) trimester pregnant and non-pregnant (unfilled circles; n=12). **(b)** Bar graph of the percentage of variance explained by the first six principle components. The data has only been processed for filtering and BMIQ-normalisation, and not adjusted for cell-type heterogeneity using the reference-free deconvolution approach (Houseman et al., 2016).


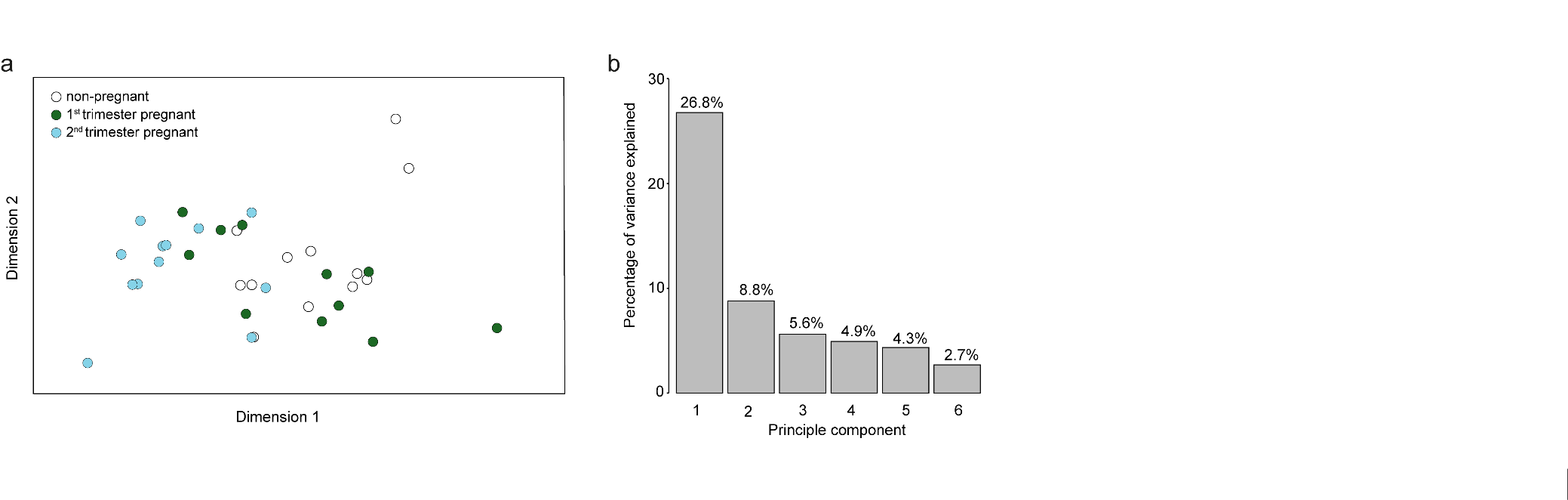


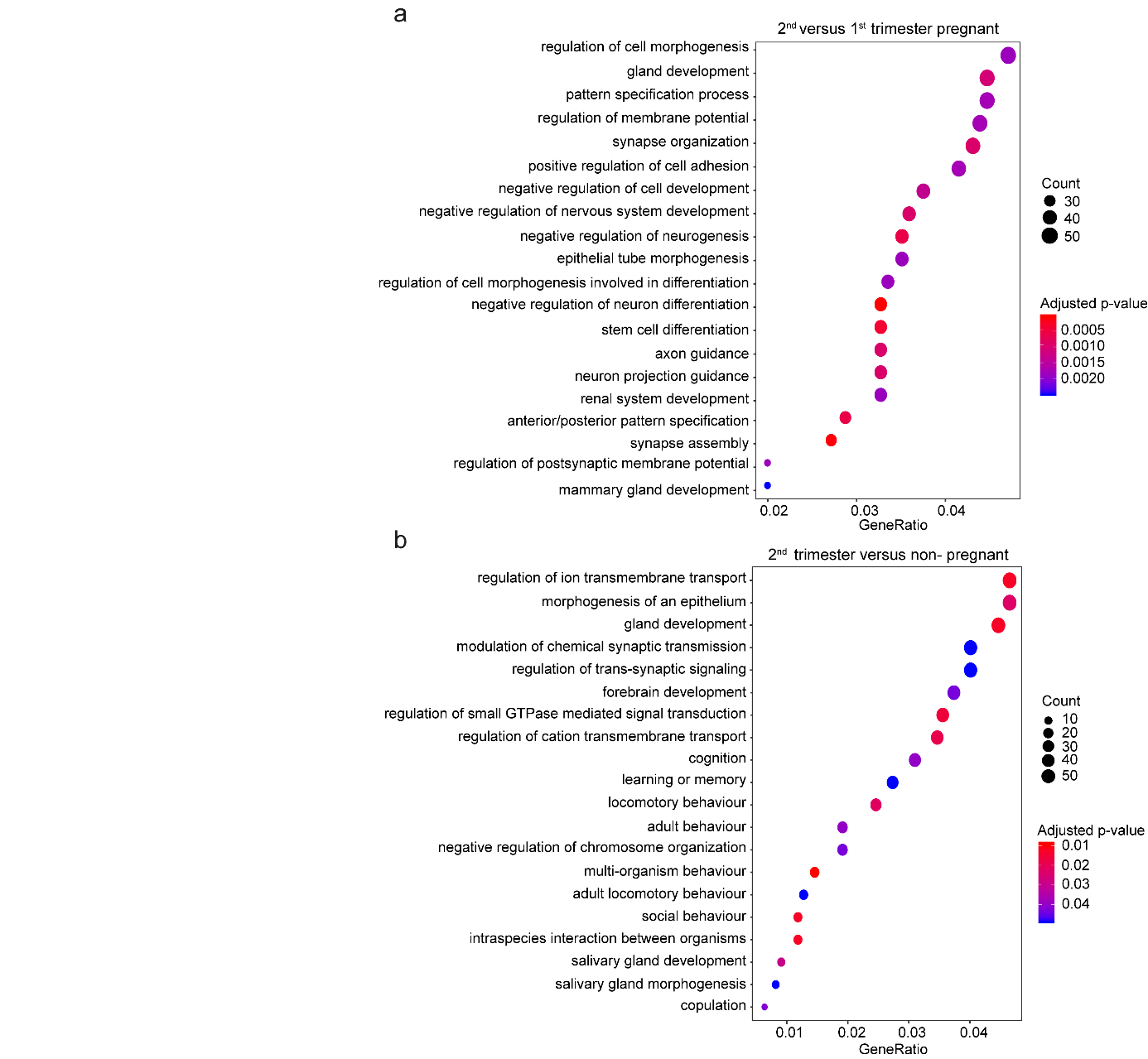
**Figure S5. Pathway analysis of differentially methylated genes during pregnancy.** Dot plots showing top 20 most significantly enriched pathways of the DMGs between **(a)** 2^nd^ and 1^st^ trimester pregnant women and **(b)** 2^nd^ trimester versus non-pregnant women. The x-axis represents the gene ratio, dot size=gene count and dot colour= adjusted p-values. Pathway analysis was done using GO. DMGs: differentially methylated genes, GO: gene ontology.
